# Drought, Climate Change, and Dryland Wheat Yield Response: An Econometric Approach

**DOI:** 10.3390/ijerph17145264

**Published:** 2020-07-21

**Authors:** Samira Shayanmehr, Shida Rastegari Henneberry, Mahmood Sabouhi Sabouni, Naser Shahnoushi Foroushani

**Affiliations:** 1 Department of Agricultural Economics, Ferdowsi University of Mashhad, Mashhad 9177948974, Iran; samira.shayanmehr@mail.um.ac.ir (S.S.); Sabouhi@um.ac.ir (M.S.S.); 2 Department of Agricultural Economics, Oklahoma State University, Stillwater, OK 74078, USA; srh@okstate.edu

**Keywords:** climate change, drought, Just and Pope, panel data, rain-fed wheat yield

## Abstract

Agriculture has been identified as one of the most vulnerable sectors affected by climate change. In the present study, we investigate the impact of climatic change on dryland wheat yield in the northwest of Iran for the future time horizon of 2041–2070. The Just and Pope production function is applied to assess the impact of climate change on dryland wheat yield and yield risk for the period of 1991–2016. The Statistical Downscaling Model (SDSM) is used to generate climate parameters from General Circulation Model (GCM) outputs. The results show that minimum temperature is negatively related to average yield in the linear model while the relationship is positive in the non-linear model. An increase in precipitation increases the mean yield in either model. The maximum temperature has a positive effect on the mean yield in the linear model, while this impact is negative in the non-linear model. Drought has an adverse impact on yield levels in both models. The results also indicate that maximum temperature, precipitation, and drought are positively related to yield variability, but minimum temperature is negatively associated with yield variability. The findings also reveal that yield variability is expected to increase in response to future climate scenarios. Given these impacts of temperature on rain-fed wheat crop and its increasing vulnerability to climatic change, policy-makers should support research into and development of wheat varieties that are resistant to temperature variations.

## 1. Introduction

One of the most challenging issues of the 21st century facing agriculture is climate change [1,2]. Studies have shown that crop production has been impacted [3] and climatic change has been found to have had a positive impact on crop production in some regions of the world, especially in agricultural areas located in latitudes above 55° N [4]. However, the negative effects of these changes are mostly found in arid and semi-arid lands [5], because these areas are located in the most fragile ecological areas, with restricted water resources. Projections of climatic change models show that global average temperature would rise between 0.5 and 3.7 °C by the end of 2100 [6,7]. Due to the mixed impacts of increased temperature and decreased precipitation, it is expected that the intensity and frequency of drought will increase [8]. An accurate understanding of how climate change impacts agriculture is expected to help policy-makers in designing appropriate policies to mitigate the adverse impacts of future climate change and to increase food security in dryland regions of the world.

Extensive studies have investigated the impacts of climate change on crop yields in different parts of the world [9,10,11,12,13,14,15,16,17,18]. For example, Gupta and Mishra [15] investigated the impact of climate change on rice production in India using a crop simulation model and Global Climate Model (GCMs) outputs. Their results show that rice yield is expected to vary from 1.2 to 8.8% (in 2006–2035), 0.7 to 12.6% (in 2036–2065), and −2.9 to 17.8% (in 2066–2095), due to climate change. De-Graft and Kweku [16] use the Just-Pope Production function to assess the impact of climatic variables on maize yield in Ghana. The findings of this research reveal that yield level is positively related to precipitation and temperature.

Iran covers an area of approximately 164.8 × 10^4^ km^2^ and is considered to be an arid and semi-arid country, with a mean annual rainfall of around 210 mm, which is less than a fourth of the average global rainfall [4]. The mean temperature in Iran is expected to rise by 1.5–4.5 °C by 2100 [19], which is expected to cause considerable changes in water resources that would have potential consequences on crop yields, particularly under rain-fed conditions [4]. Wheat is a staple food in Iran [20] and its cultivated area is around 5,437,084 ha [21]. About two-thirds of this area (3,392,336 ha) is under dryland conditions such that its production is mainly determined by rainfall and is extremely vulnerable to changes in precipitation amounts and patterns. Nevertheless, in Iran wheat yield and water returns are low because of rainfall anomalies and mismanagement of water resources [22,23]. West and East Azerbaijan provinces, located in the northwest of Iran, are among the most important in the production of dryland wheat. West Azerbaijan and East Azerbaijan are ranked third and fourth in terms of dryland wheat areas, respectively [21]. The impacts of temperature and rainfall on rain-fed wheat yield in northeastern Iran indicate that rainfall has a stronger impact on yield as compared to temperature [24]. Assessing the effects of temperature and precipitation on the yield of rain-fed wheat in the northeast of Iran shows that both climate parameters have significant effects on the output of rain-fed wheat [25]. It was also concluded that a significant correlation was detected between dryland wheat yield and rainfall during the growing season in the Khorasan province of Iran [26]. Hence, planning proper adaptation and mitigation programs is necessary for avoiding some of the adverse effects of climatic change on dryland wheat yield. Therefore, the current study focuses on assessing the impact of this change on dryland wheat yield in the northwest of Iran.

**Figure 1:**
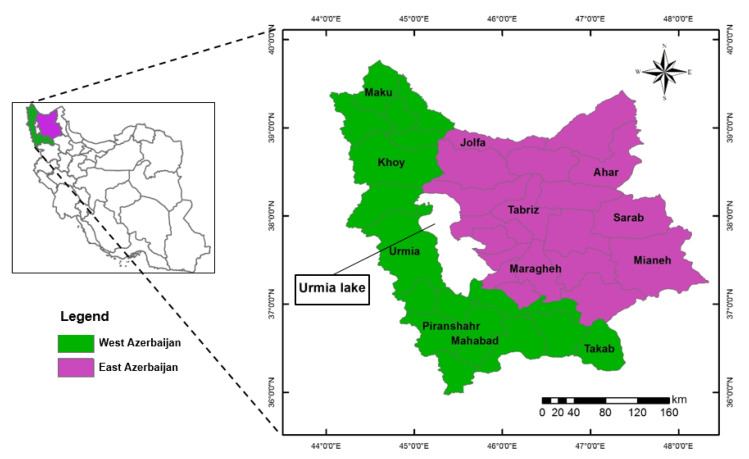
The geographical situation of the study area (West and East Azerbaijan provinces) along with the target counties.

In this area, farmers face uncertainty and stochastic threats to agricultural production, such as climate variability; these uncertainties should be addressed in production function data distribution. Under these conditions, the stochastic production function approach suggested by Just and Pope [27] is utilized to estimate the stochastic impacts of climate change on yield distributions. This approach provides a clarification of yield and yield variability, where explanatory variables affect the mean figure [1]. To the best of our knowledge, no empirical studies have applied the Just and Pope production function using panel data estimation to investigate the effect of climatic change on dryland wheat yield in northwest Iran. This is also the first study of its kind that has evaluated the impact of drought on dryland wheat yield and yield variability in Iran. On an international level, no other studies have integrated the two models of the Just and Pope stochastic production function and the statistical downscaling model (SDSM) to assess the impact of climatic change on wheat yield.

This study utilizes the Just and Pope stochastic production function approach and the SDSM model to quantify the impacts of future climate change on dryland wheat yield and yield variability in the northwest of Iran.

## 2. Materials and Methods

### 2.1. Study Area

The study area covers an area of approximately 8.26 × 10^4^ km^2^ and contains the two north-western provinces of Iran (West Azerbaijan and East Azerbaijan provinces). Geographically, it extends from latitude 36° to 40° N and longitude 44° to 48° E. The average annual maximum and minimum temperatures are 17.8 °C and 6.15 °C, respectively, and the average annual rainfall is 314.9 mm. A semi-arid climate generally dominates the northwest of Iran. The study area and location of the studied sites are represented in Figure 1. According to UNEP [28], the climate of all sites is categorized as semi-arid. The geographic and climatic features of the sites studied are summarized in Table 1. The main crops produced in this area are cereals. Among these, wheat plays a major part in production under rain-fed conditions (16.1% of Iran’s total rain-fed wheat production). Wheat is mostly cultivated as winter wheat, which is planted in autumn during October and November and is harvested during May and June. Annual average rain-fed wheat yield in the study sites is approximately 908 kg ha^−1^ [21].

**Figure 2:**
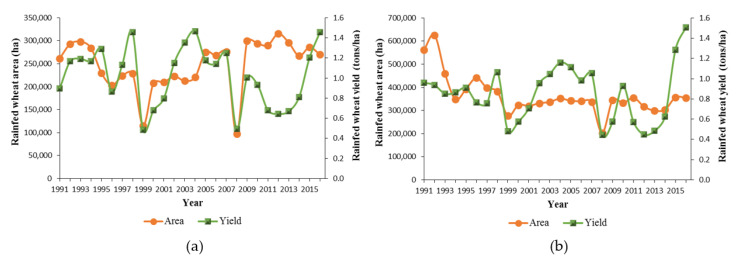
Rain-fed wheat yield in tons per hectare and per area in West Azerbaijan and East Azerbaijan Provinces. (**a**) West Azerbaijan Province. (**b**) East Azerbaijan Province.

The yield and cultivated area trends of dryland wheat in West Azerbaijan and East Azerbaijan provinces are shown in Figure 2. The graph of the area under dryland wheat cultivation in West Azerbaijan province illustrates dramatic fluctuations and a significant upward trend, with two drops in 1999 and 2008 (Figure 2a). In East Azerbaijan province, the area under dryland wheat cultivation over the years has not increased much. The cultivated area reduced in 1994 and 1999 and fluctuated until 2000 whereupon it started to indicate a steady but slow growth, with a sharp drop in 2008 (Figure 2b).

With regard to yield, Figure 2 shows that over the years there has been considerable variability in yield level. Climate variability is considered as the primary cause of yield fluctuations, year to year. As Nouri, et al. [29] argued, yield variations are significantly caused by precipitation anomalies in west and northwest Iran.

### 2.2. Data Collection

This study applies three types of data to examine the impact of climatic change on rain-fed wheat yield and yield variability: (i) data for downscaling climate change projections, (ii) data for calculating drought index and (iii) data for estimating the econometric model.

#### 2.2.1. Data for Downscaling Climate Change Projections

The daily observed data for minimum temperature (°C), maximum temperature (°C), and precipitation (mm) were obtained from Iran’s Meteorological Organization for Tabriz (the capital city of East Azerbaijan province) and Urmia (the capital city of West Azerbaijan province) synoptic stations for the period 1961–2010. The reason for choosing these stations is the existence of climatic data for the years 1961–2010. These data were employed as predictands for calibrating and validating the downscaling model. The large scale daily predictors for the second generation Canadian Earth System Model (CanESM2) were developed by the Canadian Center for Climate Modelling and Analysis (CCCma) for the Tabriz and Urmia stations. We employed these data to project future climatic parameters for Representative Concentration Pathways (RCPs: 2.6, 4.5, and 8.5). Climate scenarios have been provided for the future period of 2041–2070. The daily reanalysis data was considered as a set of observed large-scale predictors representing present climate conditions (1961–2005), which were provided from the National Center for Environmental Prediction (NCEP). These data were downloaded from the Canadian Climate Data and Scenarios website for 12 listed synoptic stations.

#### 2.2.2. Data for Calculating Drought Index

The monthly observed data for minimum temperature (°C), maximum temperature (°C), mean temperature (°C), and precipitation (mm) were obtained from Iran’s Meteorological Organization for the stations given in Table 1.

#### 2.2.3. Data for Estimating the Econometric Model

The daily observed data for minimum temperature (°C), maximum temperature (°C), and precipitation (mm) were obtained from Iran’s Meteorological Organization for 12 synoptic stations for the period 1991–2016. These data were converted to monthly mean averages of maximum and minimum temperature and a monthly average total of precipitation for the dryland wheat-growing period before analysis was done. Dryland wheat yield and cultivated area data were provided by the Ministry of Agriculture Jihad of Iran for 12 study counties for 1991–2016. The selection of the counties was constrained by the availability of data.

### 2.3. Methods

**Figure 3:**
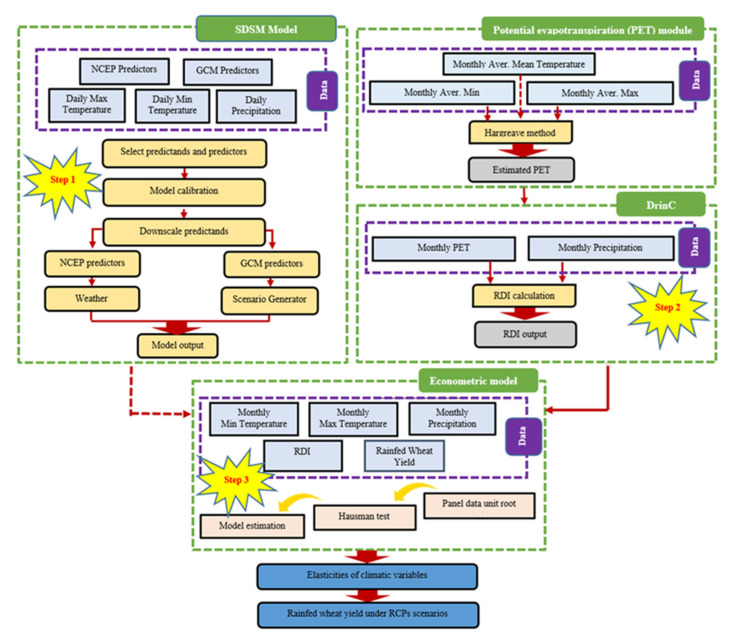
The framework of the applied methodology.

The main aim of this study was the evaluation of the impact of future climatic change on dryland wheat yield and yield variability. To reach this objective, we performed three steps. First, a statistical downscaling model was used to project the minimum and maximum temperature, and precipitation under RCP 2.6 (low emission scenario) [30], RCP 4.5 (intermediate emission scenario) [31], and RCP 8.5 (high emission scenario) [32] from 2041 to 2070 for Tabriz and Urmia stations. Second, the Reconnaissance Drought Index (RDI) was calculated using DrinC software. Finally, an econometric approach was applied to determine the relationship between climatic parameters and dryland wheat yield in order to simulate yield based on the projected climate parameters’ levels. The complete structure of the framework is presented in Figure 3.

#### 2.3.1. Step 1. Projecting Future Climatic Change Using Statistical Downscaling Model (SDSM 5.3)

GCMs are considered as the most comprehensive tools available to generate information about current and future climate for emissions scenarios. However, the GCM outputs are too coarse to capture the local climate, and therefore the downscaling technique must be used [33]. This study applied SDSM to downscale GCM-CanESM2 outputs. The CanESM2 is the only model that provides daily predictors to use directly in the SDSM model [34].

SDSM has been widely employed as an effective statistical downscaling technique in climatic studies [35]. This model has been improved by Wilby, et al. [36] and generates local climate parameters by a linear regression method and stochastic weather generator in order to make a statistical connection between local climatic variables and large-scale predictors [37]. The SDSM model contains five steps: the selection of predictand (local climatic variables) and predictor (large-scale climatic variables), model calibration, generation of weather, validation of the model, and climate scenario generation for the future [37].

##### Downscaling Daily Temperature and Precipitation Time Series

Daily precipitation and maximum and minimum temperature were chosen as predictands for the downscaling experiments. Precipitation and temperature have been measured at the Iran Meteorological Organization for 1961–2005. The large-scale predictor variables were derived from the reanalyzed NCEP data for 1961–2005. Climate variables for the future scenarios were provided from the CanESM2 location that was closest to the study area. Data were ultimately extracted for the period 2041–2070.

##### Calibration and Validation of SDSM 

The first 30 years of the study period (1961–1990) were used for calibrating the regression model, and the time series from 1991 to 2005 (the remaining 15 years of data) were utilized for SDSM validation. The performance of the SDSM was evaluated by MAE, NSE, and RMSE indicators [38] that were calculated from Equations (1) to (3):

(1) $$\mathrm{MAE}=\frac{\sum_{i=1}^{n}\left|E_{i}-M_{i}\right|}{n}$$

(2) $$\mathrm{NSE}=1-\frac{\sum_{i=1}^{n}{\left(E_{i}-M_{i}\right)}^{2}}{\sum_{i=1}^{n}{\left(M_{i}-\bar{M}\right)}^{2}}$$

(3) $$\mathrm{RMSE}=\sqrt{\frac{\sum_{i=1}^{n}{\left(E_{i}-M_{i}\right)}^{2}}{n}}$$

In the above equations, $B_{i}\;$ is the observed value, $E_{i\;}$ is the simulated value, $\bar{M}$ and $\bar{E}\;$ are the mean of the observed and simulated values, respectively, and n is the number of events.

#### 2.3.2. Step 2. Calculating the Reconnaissance Drought Index (RDI)

The Reconnaissance Drought Index (RDI) has been used for characterization and monitoring of drought based on the water deficit data. This index is an applied index for studies of drought impact on agriculture because it considers both rainfall and potential evapotranspiration (PET), which are the main parameters in plant growth [39]. The RDI index is based on the ratio of the cumulative precipitation and PET [40]. The RDI’s initial value (α_z_) is calculated for the jth month of the ith year using the following formula:

(4) $$\alpha_{z}^{\left(i\right)}=\frac{\sum_{j=1}^{z}P_{ij}}{\sum_{j=1}^{z}PET_{ij}}\;,\;i=1\left(1\right)K\;\mathrm{and}\;j=1\left(1\right)z$$

where P_ij_ is the precipitation of month j of year i, PET_ij_ refers to the potential evapotranspiration of month j of year i, and K denotes the total number of years. The mean annual values of α (${\bar{\alpha }}_{12})$ refers to the aridity index of each region [41].

The standardized form of RDI (RDI_st_) is obtained through a standardization process [42], specified as:

(5) $$RDI_{st\left(z\right)}^{\left(i\right)}=\frac{y_{z}^{\left(i\right)}-{\bar{y}}_{z}}{{\hat{\sigma }}_{yz}}$$

where y_z_ is ln($\alpha_{z}^{\left(i\right)}$), ${\bar{y}}_{z}$ is the arithmetic mean of y_z_, and ${\hat{\sigma }}_{yz}$ is its standard deviation. In this study, we applied gamma distribution for calculating RDI_st_. If the RDI values were positive, they show wet periods, while negative values show dry periods compared with the normal condition of the region. Drought severity is categorized as light dry (0 to −1); moderately dry (−1 to −1.5); severely dry (−1.5 to −2); and extremely dry (−2 or less) [43]. In the econometric model, we set the drought dummy variable equal to one for dry years (negative values of RDI) and zero otherwise.

#### 2.3.3. Step 3. Estimation Technique and Model Specification

In this study, we applied a production function approach suggested by Just and Pope [37], which determines not only the impacts of climatic parameters on the average yield but also their impacts on yield variability. This approach has been extensively applied in previous studies (e.g., Khan, et al. [44], Kumbhakar and Tsionas [45], Lien, et al. [46], Asche, et al. [47], Isik and Khanna [48], Ogundari and Akinbogun [49], and Chen, et al. [50]). Before fitting the data into the Just and Pope model, the stationarity of the variables is examined using panel unit root tests. In this study, we carried out the Levin, Lin, and Chu (LLC), ADF-Fisher-type, and Breitung panel unit root tests. In the next step, the Breusch and Pagan [51] and White [52] tests, with a null hypothesis of homoscedasticity, are used to examine whether any significant yield variance is observable [12,53].

The Just and Pope Production function utilized in the current study has the following specification:

(6) y=f(X,α)+k(X,β)ε

where y is the dryland wheat yield, and X is a set of independent variables (rain-fed wheat growing season maximum and minimum temperature, rain-fed wheat growing season precipitation, rain-fed wheat growing season drought, the cultivated area, and time trend). Function f describes how mean yield is influenced by changes in X, with α as the associated vector of estimated parameters. The function k also provides the way in which X influences the yield variance, with β as the corresponding vector of estimated parameters. Finally, ε is an exogenous production shock with mean zero and unity variance.

The model can be estimated by two different procedures: (1) the maximum likelihood estimation (MLE); (2) three-step feasible generalized least squares (FGLS) [27]. Most previous studies have applied the FGLS procedure, but the MLE procedure is more efficient and less biased than FGLS estimations in the case of small samples [54]. Given the large sample in the current study, the FGLS approach was utilized to estimate models.

The following essential steps are involved in estimating the Just-Pope using FGLS:

First, we estimate Equation (7) using a panel data model, and obtain the residual ν.

(7) y=f(X, α)+v

Second, we regress the logarithm of the squared residuals of the estimated equation on X, as in Equation (8).

(8) $$\ln\left({\hat{v}}^{2}\right)=k\left(X,\;\beta \right)+\varepsilon$$

These are consistent estimates of the variances. Finally, we use the antilogarithms’ predicted values of $\ln\left({\hat{v}}^{2}\right)$ as weights to modify Equation (7), removing the effect of heteroscedasticity to get a final estimate of α. This is done using Equation (9).

(9) $$yk^{-0.5}\left(X,\hat{\beta }\right)=f\left(X,\alpha \right)k^{-0.5}\left(X,\hat{\beta }\right)+vk^{-0.5}\left(X,\hat{\beta }\right)$$

Then alpha informs us as to how a change in X influences mean yield. Previous empirical studies of production have applied either a linear functional form of the independent variables (e.g. Chen, McCarl and Schimmelpfennig [50], Arshad, et al. [55], Mahmood, et al. [56]) or a non-linear form (e.g., Horowitz [57], Sarker, Alam and Gow [54], Sarker, Alam and Gow [11], Isik and Devadoss [58]). Hence, in this study, the Just-Pope approaches with both linear and nonlinear (i.e. quadratic functional form) are estimated. All analyses of the specified Just and Pope function are conducted using Stata v.16.0. The mean yield production function is represented as:

Non-linear

(10) $$\begin{array}{ll} f\left(x,\alpha \right)= & \beta_{0}+\beta_{t}\mathrm{Trend}+\beta_{p}\mathrm{Drought}+\beta_{u}\mathrm{Area}+\overset{3}{\underset{j=1}{\sum }}\beta_{1j}x_{j}+\overset{3}{\underset{j=1}{\sum }}\beta_{2j}x_{j}^{2}\\ & \;+\overset{3}{\underset{j=1}{\sum }}\overset{3}{\underset{h\left(h\neq j\right)=1}{\sum }}\beta_{jh}x_{j}x_{h} \end{array}$$

Linear

(11) $$f\left(x,\alpha \right)=\beta_{0}+\beta_{t}\mathrm{Trend}+\beta_{p}\mathrm{Drought}+\beta_{u}\mathrm{Area}+\overset{3}{\underset{j=1}{\sum }}\beta_{1j}x_{j}$$

where Trend represents the time trend, Drought is the drought dummy variable, Area is cultivated area, x_j_ and x_h_ are explanatory variables that involve climate variables during the dryland wheat-growing months in a year, and β’s imply coefficients to be estimated. The inclusion of time trend represents technological progress in the agricultural sector during the studied periods. 

For this study, the risk equation is specified based on Harvey [59]. The variance function k(X,β) is assumed to have exponential form:

Non-linear

(12) $$\begin{array}{ll} k\left(X,\beta \right)=\exp & (\gamma_{0}+\gamma_{t}\mathrm{Trend}+\gamma_{p}\mathrm{Drought}+\gamma_{u}\mathrm{Area}+\overset{3}{\underset{j=1}{\sum }}\gamma_{1j}x_{j}\\ & \;+\overset{3}{\underset{j=1}{\sum }}\gamma_{2j}x_{j}^{2}+\overset{3}{\underset{j=1}{\sum }}\overset{3}{\underset{h\left(h\neq j\right)=1}{\sum }}\beta \gamma_{jh}x_{j}x_{h}) \end{array}$$

Linear

(13) $$k\left(X,\beta \right)=\exp(\gamma_{0}+\gamma_{t}\mathrm{Trend}+\gamma_{p}\mathrm{Drought}+\gamma_{u}\mathrm{Area}+\overset{3}{\underset{j=1}{\sum }}\gamma_{1j}x_{j})$$

where Trend represents the time trend, Drought is the drought dummy variable, Area is cultivated area, x_j_ are explanatory variables that involve climate variables during the dryland wheat-growing months in a year, and γ’s imply coefficients to be estimated. 

## 3. Results and Discussion

First, we looked at the results of the SDSM for the study area. Second, the RDI index was calculated. Then, the production function estimations were analyzed. Finally, using previous steps’ results, we forecasted the impacts of future climate change on mean yield and yield variability.

### 3.1. Statistical Downscaling Model

SDSM 5.3 was utilized to evaluate the impact of local climate change based on a statistical downscaling technique. Five main steps were performed: the selection of predictors, calibration of the model, generation of weather, validation of the model, and climate scenario generation. 

#### 3.1.1. Selection of Predictors

The choice of predictors is a critical step in applying SDSM. The daily NCEP data for the period 1961–2005 was applied to determine the predictor variables. In this study, the procedure of selecting appropriate predictors is based on [34,36]. The final set of predictor variables for stations is as follows: 

Maximum temperature: mslp, p500, prcpgl, s500gl, s850gl, shumgl, and tempgl. 

Minimum temperature: mslp, p500, s850gl, and tempgl.

Precipitation: mslp, p5u, p5-z, p500, p850, p8zhgl, prcpgl, and s850gl. 

#### 3.1.2. SDSM Performance

**Figure 4:**
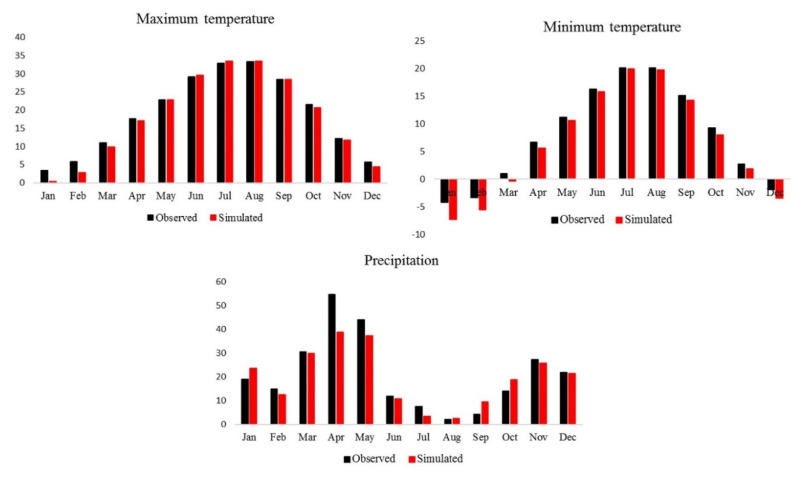
Validation of Statistical Downscaling Model (SDSM) performance for minimum temperature, maximum temperature, and precipitation by comparing the monthly mean for the observed and simulated data for Tabriz station during 1991–2005.

The calibration module in SDSM was used for 1961–1990, then the weather-generator module was applied for SDSM validation. The observed data and results of the climate simulation were then compared by summary and frequency analysis in the SDSM model for the period 1991–2005, as is shown in Figure 4. Investigations of the monthly mean precipitation, maximum temperature, and minimum temperature showed a good agreement between the observed and simulated data for minimum and maximum temperatures that were similar. However, precipitation data deferred more, particularly in April. This may be due to missing observed data on precipitation that adversely affected the performance of the model [34].

SDSM performance was evaluated by RMSE, NSE, and MAE indicators (see Table 2). The evaluation of these criteria revealed that the SDSM performed well for downscaling the maximum temperature, minimum temperature, and precipitation.

The lower values of MAE (0.2–1.1) and RMSE (0.18–1.4), and upper values of NSE (>0.80) for the validation period indicated that the simulated data of maximum temperature, minimum temperature, and precipitation were acceptable.

#### 3.1.3. Projection of Precipitation and Temperature

In the next step, the future climatic parameters simulated by CanESM2 were downscaled. These, under RCP 2.6, 4.5, and 8.5, were analysed for 2041–2070. Then, in order to identify the consequences of climatic change on the dryland wheat yield, climatic parameters of the dryland wheat-growth period were evaluated for 2041–2070. Finally, these parameters were compared with the baseline period (1981–2011). The percentage changes in maximum temperature, minimum temperature, and precipitation of dryland wheat-growing period under different RCPs for 2041–2070 are reported in Table 3.

### 3.2. The Reconnaissance Drought Index (RDI)

**Figure 5:**
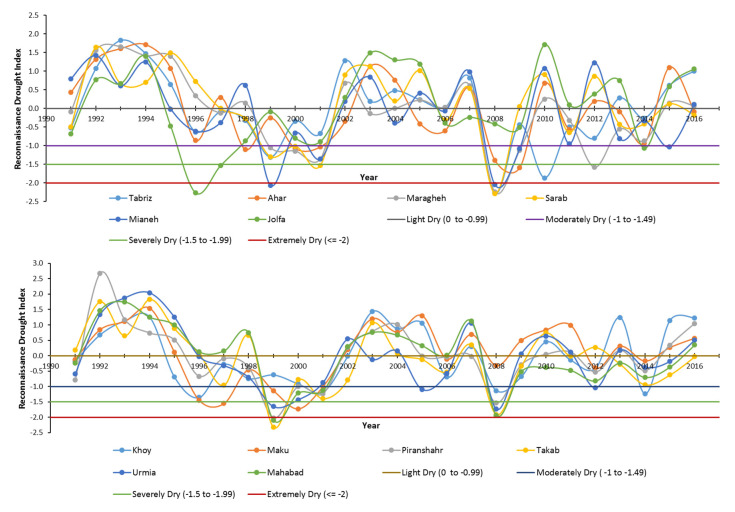
The wheat growing season Reconnaissance Drought Index (RDIst) graph (Oct–Jun) for target counties of East Azerbaijan and West Azerbaijan provinces.

Climatic data on monthly maximum temperature, monthly minimum temperature, and monthly precipitation were provided from Iran’s Meteorological Organization for study sites during 1991–2016. We estimated the potential evapotranspiration using the Hargreaves method [60] and then calculated the RDI using the DrinC software [61]. The wheat-growing period drought conditions (Oct-Jun) for study sites, based on RDI_st_ classification, are shown in Figure 5. As can be seen, all sites experienced drought conditions (the negative RDI values) during this period. The severely and extremely dry years occurred in 1996, 1999, and 2008. It should be noted that, in the econometric model, we set the drought dummy variable equal to one for dry years (negative values of RDI) and zero otherwise.

### 3.3. Pre-estimation Specification Tests

The first step in the analysis is to examine the stationary of variables. For this purpose, we utilized the Levin, Lin, and Chu (LLC), ADF-Fisher-type, and Breitung panel unit root tests. As reported in Table 4, the null hypothesis of unit roots is rejected with a 99% confidence level for all variables in the model. Therefore, all variables under the model are stationary.

In the next step, we performed the White and Breusch-Pagan-Godfrey heteroscedasticity tests to assess homoscedasticity. As can be seen in Table 5, the null hypothesis of homoscedasticity was rejected with a 99% confidence level. The presence of heteroscedasticity for the model directed us to proceed with the Just and Pope estimation technique.

The random-effect model and fixed-effect model are employed for panel data. The Hausman test is utilized to determine which should be applied. As Table 5 shows, the null hypothesis of no correlation between regional effects and independent variables is rejected. Consequently, a fixed-effect model with regional specific effects is applied.

### 3.4. Impact of Climate Change and Drought on Average Yield and Yield Variability of Dryland Wheat (Linear Model)

The results of average yield and yield variability estimations of dryland wheat for the linear model are demonstrated in Table 6. The first part of this table reports the coefficient of the mean yield function and the second part reports coefficient of the yield risk function. In mean yield function, the overall F statistics has a *p*-value of 0.000 and implies that independent variables are jointly significant. The results indicate that precipitation and maximum temperature have a positive and significant impact on the yield. The negative sign on minimum temperature shows that a higher average of minimum temperature decreases the dryland wheat yield. The negative relationship between mean yield and minimum temperature uncovered by this study is consistent with the findings of Gupta, et al. [62], and Lobell, et al. [63]. The drought dummy variable is negatively and significantly related to the mean yield. The cultivated area variable is also negatively and significantly associated with the average yield. Similar findings are presented by studies conducted in Pakistan and the U.S. [50,55]. Therefore, an increase in dryland wheat cultivated area is assumed to decline the mean yield because more marginal land is brought into production with the negative effect increasing in absolute terms along with the cultivated area. Technological advances as identified by a time trend variable increased average yield as expected. This result indicated that dryland wheat yield increases over time because of technological progress including new crop varieties, development irrigation coverage, and increased use of fertilizer. The result was in agreement with the findings of Chen, McCarl and Schimmelpfennig [50], and Poudel, et al. [64].

In terms of yield variability, the regression coefficients for yield variance reveal that an increase in maximum temperature contributes positively and significantly to yield risk. Also, precipitation has a positive effect on yield variability. These findings reveal that precipitation and maximum temperature are risk-increasing inputs. On the contrary, the relationship between minimum temperature and yield variability is negative. The drought dummy variable is regarded as a risk-increasing factor. The cultivated area variable has a negative and significant impact on yield variance. Therefore, the cultivated area variable is considered as a risk-decreasing factor. Finally, the time trend variable has a positive and significant impact on yield variability. This result is in line with the findings of Chen, McCarl and Schimmelpfennig [50], and Poudel, Chen and Huang [64].

### 3.5. Impact of Climate Change and Drought on Average Yield and Yield Variability of Dryland Wheat (Non-linear Model)

Table 7 presents the findings of mean yield and yield variability for dryland wheat with non-linear (quadratic) form. The results indicate that minimum temperature has a negative and significant impact on the mean yield. The positive sign on the maximum temperature and precipitation shows that a higher mean of maximum temperature and precipitation decreases dryland wheat yield. Both drought and planted areas are negatively related to mean yield. Though the effect of drought is significant, that of the cultivated area is not significant. Quadratic terms for all climatic variables are significant with a negative effect on average yield. The interaction of maximum temperature with minimum temperature has a positive and significant impact on average yield while the other interaction terms have a positive and insignificant effect.

From the viewpoint of the yield variability, as can be seen in Table 7, the impact of maximum temperature on dryland wheat yield variability is positive and significant. The minimum temperature is statistically significant with a negative impact on yield variability, while precipitation has a positive effect on yield variability. These results imply that maximum temperature and precipitation are the risk-increasing inputs and minimum temperature is a risk-decreasing input. Increasing dryland wheat planted area decreases yield variability, while increasing drought conditions and technological advances are considered as risk-increasing factors. Quadratic terms for maximum temperature are significant with a negative effect on yield variability. The other quadratic terms have a negative and insignificant effect on yield variability. The interaction of maximum temperature and precipitation has a negative impact on yield variability while the remaining interaction terms have a positive effect.

### 3.6. Elasticities of Climatic Variables

Since the non-linear model has quadratic and interaction terms, it is impossible to contrast the extent and signs of the estimated coefficient in the non-linear model to those in the linear model. The estimation of elasticity that gives a common denominator is utilized to evaluate and compare the impact of climatic parameters in the linear model and non-linear model [58]. Estimated elasticities of climatic variables for both models are reported in Table 8.

The elasticity values indicate that an increase in minimum temperature decreases the dryland wheat yield level and the yield variability in the linear model. Elasticity values in mean yield (−0.34) and yield variability (−0.88) are less than one and thus are inelastic. A 1% increase in maximum temperature increases the mean yield and yield variability by about 1.58% and 3.87%, respectively, in the linear model. Also, precipitation increases the yield and its variability in the linear model. This confirms that maximum temperature and precipitation are risk-increasing inputs and minimum temperature is a risk-decreasing input in the linear model.

The Estimated elasticities in the non-linear model reveal that a one percent increase in maximum temperature decreases mean yield by 0.32% and increases yield variability by 3.03%. The minimum temperature is a yield-increasing and a risk-decreasing input in the non-linear model. An increase in precipitation increases the average yield and its variability in the non-linear model. Therefore, precipitation is a risk-increasing input in the non-linear model.

### 3.7. Predicting the Mean Yield and Yield Variability in the Presence of Future Climatic Change

The changes in dryland wheat yield and yield variability in the presence of climate change are gauged by the findings presented in the preceding sections of this paper (percentage changes in climate variables and estimated elasticities). The findings imply that under the linear model the mean yield for both provinces would rise in response to three climatic scenarios (Table 9). East Azerbaijan province is prone to experience an increase in mean yield of around 5.81%, 3.97%, and 7.65% under RCP 2.6, RCP 4.5, and RCP 8.5, respectively, by 2070. The average yield in West Azerbaijan province is expected to increase by a maximum of approximately 5.17% in response to future climate scenarios by the end of this period. Results of the non-linear model show that, under RCP 2.6 and RCP 8.5, the mean yield for East Azerbaijan province would continue to increase in the 2041–2070 period with respect to the base period, while under RCP 4.5 the mean yield for this province is expected to reduce by about 0.40%. In West Azerbaijan province, RCP 4.5 scenario would increase the mean yield by 1.87% and other scenarios would decrease the mean yield by the end of 2070. From the viewpoint of yield variability, future climate change would increase this in the 2041–2070 period, with respect to the base period, in either model. As can be seen in Table 9, East Azerbaijan and West Azerbaijan provinces are expected to experience an increase in yield variability maximum of about 24% and 15%, respectively.

## 4. Conclusions

The CO_2_-induced climatic scenarios used in this study forecast a rise in maximum and minimum temperatures and changes in precipitation levels, which will have impacts on crop yield and yield variability. In this study, the impacts of climate change on dryland wheat yield and yield variability was investigated in the northwest of Iran. The results indicate that minimum temperature is negatively related to average yield in the linear model while the relationship is positive in the non-linear model. An increase in precipitation increases the mean yield in either model. The maximum temperature has a positive effect on the mean yield in the linear model, while this impact is negative in the non-linear model. Elasticity values under the yield variability function reveal that maximum temperature and precipitation are positively related to yield variability. The effect of the minimum temperature on yield variability is negative. This confirms that minimum temperature is a risk-decreasing input for dryland wheat. Finally, the findings reveal that future climate change is expected to increase the variance of dryland wheat yield. 

The findings of this study are expected to provide an understanding of how climate change affects dryland wheat yield and yield variability in East and West Azerbaijan provinces in Iran. Future research could focus on assessing the climatic change impact on the yield of other crops and evaluating the level of changes occurring in the allocations of agricultural land among different crops in response to climate change.

## Figures and Tables

**Table 1:** Latitude (Lat), longitude (Lon), altitude (Alt), average annual maximum (Max) and average annual minimum temperatures (Min), and total annual precipitation (Pre) for the study sites during 1991–2016.

Synoptic Stations	Lat (°N)	Lon (°E)	Alt (m)	Max (°C)	Min (°C)	Pre (mm)
**East Azerbaijan Province**						
Ahar	38.43	47.07	1391	16.7	5.4	288
Jolfa	38.93	45.60	736	21.1	10.5	217
Maragheh	37.35	46.15	1344	19.1	8.0	283
Mianeh	37.45	47.70	1110	20.8	7.5	274
Sarab	37.93	47.53	1682	16.2	1.4	250
Tabriz	38.12	46.24	1361	19.0	7.7	246
**West Azerbaijan Province**						
Khoy	38.56	45.00	1103	19.2	6.1	265
Mahabad	36.75	45.72	1351	19.5	7.0	402
Maku	39.38	44.39	1411	15.9	5.6	312
Piranshahr	36.70	45.15	1443	18.5	7.0	666
Takab	36.40	47.10	1817	16.6	2.7	316
Urmia	37.66	45.06	1328	18.1	5.2	310

**Table 2:** Results of SDSM performance for the observed and simulated data in the validation period (1991–2005).

Station Name	Climatic Factors	MAE	RMSE	NSE
Tabriz station	Maximum temperature	0.95	1.35	0.98
(East Azerbaijan province)	Minimum temperature	1.15	1.40	0.97
	Precipitation	0.12	0.18	0.86
Urmia station	Maximum temperature	0.50	0.71	0.99
(West Azerbaijan province)	Minimum temperature	0.63	0.87	0.98
	Precipitation	0.20	0.30	0.80

**Table 3:** Percentage change in climate parameters of dryland wheat-growing period for 2041–2070.

Synoptic Station	RCPs	Change in Climate Variables (%)		
		Maximum Temperature	Minimum Temperature	Precipitation
Tabriz station	2.6	2.26	1.60	7.75
(East Azerbaijan province)	4.5	2.61	1.62	1.10
	8.5	2.32	2.48	13.42
Urmia station	2.6	3.02	4.39	1.74
(West Azerbaijan province)	4.5	3.94	11.58	8.02
	8.5	3.84	11.26	2.97

**Table 4:** Unit root test results.

Variables	Fisher-ADF	LLC	Breitung
Yield (tons/ha)	109.872 ***	−3.397 ***	−7.226 ***
Area (ha)	93.649 ***	−4.480 ***	−3.953 ***
Maximum temperature (°C)	116.463 ***	−5.200 ***	−8.807 ***
Minimum temperature (°C)	119.961 ***	−6.836 ***	−6.481 ***
Precipitation (mm)	133.793 ***	−4.704 ***	−8.177 ***

Note: *** indicates rejection of the unit root hypothesis at the 1% significance level.

**Table 5:** Panel data model specification tests.

Heteroscedasticity Tests		Fixed Effects Versus Random Effects
White’s Test	Breusch-Pagan Test	Hausman Test
167.74 ***	61.59 ***	17.53 ***

Note: *** indicates significant at the 1% level.

**Table 6:** Estimates of the impact of climatic variables on average yield and yield variability of dryland wheat, using the Just and Pope linear model.

Variables	Mean Yield		Yield Variability	
	Coefficient	Standard Error	Coefficient	Standard Error
Constant	−4.240 **	2.021	−10.290 ***	2.704
Trend	0.015 **	0.007	0.037 **	0.018
Area	−0.000004 **	0.000002	−0.020 **	0.010
Drought	−0.152 **	0.065	0.036	0.363
Maximum temperature	0.100 ***	0.029	0.532 ***	0.210
Minimum temperature	−0.112 ***	0.040	−0.562 ***	0.237
Precipitation	0.015 ***	0.002	0.021	0.019
**Model statistics**				
F-test	68.38		3.625	
Prob > F	0.000		0.001	
R-squared	0.715		0.113	
Adj R-squared	0.6988		0.062	
Log-likelihood	−616.556		−658.943	
AIC	1247.113		1331.888	
BIC	1273.315		1358.089	
No. of obs.	312		312	

**Note:** ** and *** indicate significant at the 5% and 1% levels, respectively.

**Table 7:** Estimates of the impact of climatic variables on average yield and yield variability of dryland wheat, using the Just and Pope non-linear (quadratic) model.

Variables	Mean Yield		Yield Variability	
	Coefficient	Standard Error	Coefficient	Standard Error
Constant	−0.341	0.662	−57.303 **	25.034
Trend	0.002	0.002	0.012	0.022
Area	−0.000001	0.000001	−0.000008	0.00001
Drought	−0.103 **	0.050	0.202	0.464
Maximum temperature	0.210 ***	0.047	7.863 **	3.594
Minimum temperature	−0.417 ***	0.112	−5.582 *	3.061
Precipitation	0.008	0.015	0.237	0.243
Maximum temperature, squared	−0.012 ***	0.003	−0.289 **	0.129
Minimum temperature, squared	−0.017 *	0.009	−0.123	0.115
Precipitation, squared	−0.0001 ***	0.00004	−0.0005	0.0005
Maximum temperature * Minimum temperature	0.034 ***	0.010	0.389 *	0.222
Maximum temperature * Precipitation	0.0005	0.001	−0.014	0.017
Minimum temperature * Precipitation	0.0008	0.001	0.014	0.015
**Model statistics**				
F-test	44.68		0.99	
Prob > F	0.000		0.459	
R-squared	0.738		0.039	
Adj R-squared	0.717		−0.037	
Log-likelihood	−621.255		−701.877	
AIC	1268.511		1429.755	
BIC	1317.170		1478.414	
No. of obs.	312		312	

Note: *, **, and *** indicate significant at the 10%, 5%, and 1% levels, respectively.

**Table 8:** Elasticities of climate variables for the linear and non-linear models.

Functional Form	Climate Variables	Average Yield	Yield Variability
Linear	Maximum temperature	1.581	3.876
	Minimum temperature	−0.346	−0.885
	Precipitation	0.366	0.997
Non-linear	Maximum temperature	−0.328	3.032
	Minimum temperature	0.001	−0.671
	Precipitation	0.396	1.421

**Table 9:** Percentage changes in average yield and yield variability for East Azerbaijan and West Azerbaijan provinces under RCP 2.6, RCP 4.5, and RCP 8.5 for the period of 2041–2070, as compared to the base period.

Provinces	Functional Form	RCP	Average Yield	Yield Variability
East Azerbaijan province	Linear	2.6	5.81	15.00
		4.5	3.97	9.79
		8.5	7.65	20.09
	Non-linear	2.6	2.30	16.68
		4.5	−0.40	8.38
		8.5	4.49	24.29
West Azerbaijan province	Linear	2.6	3.90	9.56
		4.5	5.17	13.02
		8.5	3.30	7.91
	Non-linear	2.6	−0.28	8.66
		4.5	1.87	15.48
		8.5	−0.05	8.27
